# Bacterial Quorum-Sensing Systems and Their Role in Intestinal Bacteria-Host Crosstalk

**DOI:** 10.3389/fmicb.2021.611413

**Published:** 2021-01-28

**Authors:** Liang Wu, Yubin Luo

**Affiliations:** Department of Rheumatology and Immunology, Frontiers Science Center for Disease-Related Molecular Network, West China Hospital, Institute of Immunology and Inflammation, Sichuan University, Chengdu, China

**Keywords:** bacterial communication, quorum sensing, autoinducers, metabolites, host cells

## Abstract

Quorum-sensing (QS) system is a rapidly developing field in which we are gradually expanding our understanding about how bacteria communicate with each other and regulate their activities in bacterial sociality. In addition to collectively modifying bacterial behavior, QS-related autoinducers may also be embedded in the crosstalk between host and parasitic microbes. In this review, we summarize current studies on QS in the intestinal microbiome field and its potential role in maintaining homeostasis under physiological conditions. Additionally, we outline the canonical autoinducers and their related QS signal-response systems by which several pathogens interact with the host under pathological conditions, with the goal of better understanding intestinal bacterial sociality and facilitating novel antimicrobial therapeutic strategies.

## Introduction

Microorganisms are omnipresent in nature and form a significant part of our micro- and macro-environment. The human body is home to several microorganisms, especially at mucosal sites. The first studies suggestive of quorum-sensing (QS) as a mode of cell to cell communication within bacterial communities were published in 1965 (Bassler and Losick, 2006). Prior to this, researchers focused on eliminating bacteria using conventional antimicrobials. In the 1950s, success in rescuing *Clostridium difficile* infection using fecal microbiota transplantation (FMT) introduced a new perspective, i.e., introduction of a mixed microbial community, instead of eliminating specific bacteria, may benefit the host in fighting microbial infection. Around the same time, a phenomenon of intercellular communication in specific bacterial communities was discovered (Tomasz, 1965; Bassler and Losick, 2006). Researchers began to realize that gregarious communication is common in bacterial sociality, and named it the QS system (Bassler and Losick, 2006).

QS is a cell-cell communication process that enables bacteria to feel their surrounding environment and to regulate their density and behavior, which allows them to live like multicellular organisms (Bassler and Losick, 2006). They make use of their community to manage self-competition, as well as collectively interact with their host. Gut microbiomes can be identified and characterized from feces or other samples using 16S ribosomal RNA (rRNA) gene sequencing technology. Here, we summarize the classical QS systems in intestinal bacteria and highlight the newly discovered functions mediated by QS. Moreover, the potential roles of QS in host-bacterial interaction are also discussed. It might provide a new perspective for microbiome researchers to advance toward development of new medicines to treat devastating microbial infection and associated pathologies.

## Classical QS in Bacterial Communication

**FIGURE 1:**
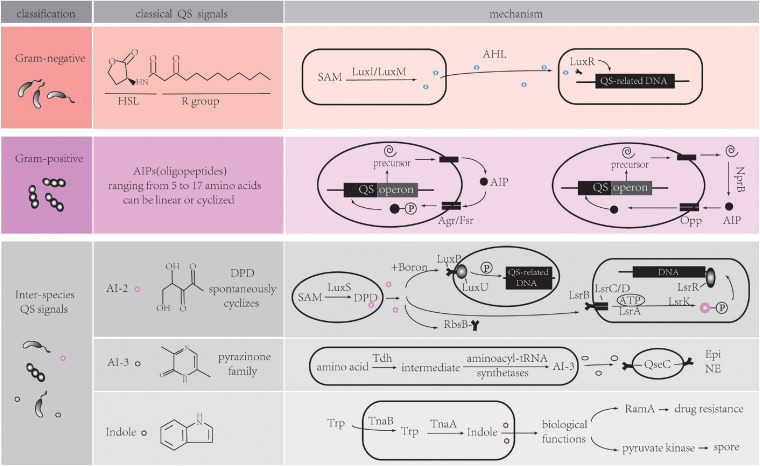
Five main signaling molecules in QS system. HSL, homoserine lactone; SAM, S-adenosylmethionine; AHL, acyl-homoserine lactones; AIP, autoinducing Peptides; NprB, neutral protease B; Opp, oligopeptide permease system; AI, autoinducer; DPD, 4,5-dihydroxy-2,3-pentanedione; Tdh, threonine dehydrogenase; Epi, epinephrine; NE, norepinephrine; Trp, tryptophan; Tna, tryptophanase. Detailed information can be found in section “Classical QS in Bacterial Communication.”

QS is defined as a bacterial cell-cell communication process that controls bioluminescence, competence, antibiotic production, sporulation, biofilm formation, and virulence factor secretion in bacteria. Recent research has shown that the scope of QS extends to inter-kingdom communication, mediated by several newly identified extra-cellular signaling molecules called autoinducers (AIs). Among these AIs, five main signaling molecules are involved in the classical QS system (Figure 1).

### Acyl-Homoserine Lactones (AHL) QS System in Gram-Negative Bacteria

Both Gram-positive (Gram^+^) and Gram-negative (Gram^–^) bacteria use QS to communicate with each other. Although the types of QS pathways are different in Gram^+^ and Gram^–^ bacteria, they all have fundamental biological roles. Gram^–^ QS system has several common features (Ng and Bassler, 2009; Papenfort and Bassler, 2016). Firstly, AIs are molecules synthesized from S-adenosylmethionine (SAM) as a substrate. Acyl-homoserine lactones (AHLs) are the most common class of AIs. They have an N-acylated homoserine-lactone ring as the core and a 4–18 carbon acyl chain containing modifications. The length of the acyl chain affects their stability (von Bodman et al., 2008; Galloway et al., 2011). LuxI-type enzymes are the major, but not the sole producer of AHLs. A LuxM synthase found in *Vibrio harveyi*, which is not a homolog of LuxI, can produce AHLs for their intra-species communication (Bassler et al., 1993). SAM can be metabolized into special signals which can be sensed by different bacteria species. Diffusible signal factor (DSF) type molecules are synthesized by RpfF proteins in *Pseudomonas aeruginosa* and *Burkholderia cenocepacia* (Ryan et al., 2015; Zhou et al., 2015). Cholera autoinducer 1 (CAI-1) is produced by the CAI-1 AI synthase (CqsA) in *Vibrio cholerae*. Due to the common prevalence of homologs of CqsA in *Vibrio* spp., various CAI-1 are produced by this bacterial species. Additionally, *Vibrio* spp. may have different affinities to CAI-1 not produced by themselves, suggesting CAI-1 is a vibrio inter-genus communication molecule (Miller et al., 2002; Higgins et al., 2007; Rutherford and Bassler, 2012; Papenfort and Bassler, 2016).

Secondly, AIs bind to specific membrane receptors or cytoplasmic proteins (Papenfort and Bassler, 2016). LuxR-type receptors, the cytoplasmic transcription factors, detect freely diffusible AHLs in cytoplasm and bind cognate AHLs. The stable LuxR-AHL complexes can bind to DNA while unbound LuxR proteins are rapidly degraded (Zhu and Winans, 2001; Rutherford and Bassler, 2012; Figure 1, Gram negative part). These LuxR/LuxI-type systems, including LasR/LasI and RhlR/RhlI in *Pseudomonas aeruginosa*, mediate inter-cellular communication (Papenfort and Bassler, 2016). Some annotated LuxR proteins belong to LuxR-solo receptor or orphan LuxR class. In the absence of LuxI synthases, they detect different AHL molecules produced by other bacterial species, thus mediating inter-species communication. Examples of these are QscR in *P. aeruginosa* (Zhu and Winans, 2001; Hudaiberdiev et al., 2015; Papenfort and Bassler, 2016), SdiA (LuxR homolog) in *Escherichia*, which can also respond to mammalian host-produced small molecules (Hughes et al., 2010; Nguyen et al., 2015; Whiteley et al., 2017).

Thirdly, combined receptors work as transcription factors to regulate dozens to hundreds of genes that affect biofilm formation, virulence, and other biological processes in bacteria. QS molecule receptors establish a feed-forward loop when regulating genes expression, which is called autoinduction. This mechanism increases the autoinducers synthesis, consequently promoting synchronous genes expression in the population (Papenfort and Bassler, 2016).

### Autoinducing Peptides (AIP) QS System in Gram-Positive Bacteria

While QS circuits share some common features, there are stark distinctions between Gram^+^ and Gram^–^ bacteria. The AIs in many Gram^+^ bacteria are oligopeptides (AIPs). There are two kinds of canonical AIP-QS circuits. One class of AIPs is encoded as a precursor from QS operon, then processed and secreted extracellularly by specialized transporters. The AIPs ranging from 5 to 17 amino acids can be linear or cyclized (Okada et al., 2005; Bouillaut et al., 2008; Thoendel et al., 2011; Rutherford and Bassler, 2012). Membrane-bound, two-component sensor histidine kinases, such as Agr system in *Streptococcus aureus*, and Fsr system in *Enterococcus faecalis* serve as AIP receptors (Waters and Bassler, 2005; Zschiedrich et al., 2016; Ali et al., 2017). The sensor kinases auto-phosphorylate after binding to AIPs, and the phosphoryl group is delivered to a cognate cytoplasmic response-regulator protein that controls the expression of QS-related genes (Rutherford and Bassler, 2012). In *S. aureus*, AIPs are variable and have coevolved with their receptors. Non-cognate AIPs have an inhibitory effect on QS in other strains, allowing one strain to establish its specific niche (Lyon et al., 2002; Geisinger et al., 2009).

In other canonical AIP-QS circuits, pre-AIPs are secreted by the secretory system and processed by extracellular proteases such as secreted neutral protease B (NprB). AIPs are imported and go on to bind transcription factors to regulate DNA expression through the oligopeptide permease system (Opp) (Gominet et al., 2001; Pomerantsev et al., 2009; Figure 1, Gram positive part). The PapR-PlcR system in *Bacillus cereus* is an example of a typical QS that functions in this way (Slamti and Lereclus, 2002). In addition, there is autoinduction that results from transcription of the QS operon which encodes pre-AIPs, transporters, receptors, regulators and proteases, leading to the synchronization of QS response (Gominet et al., 2001; Pomerantsev et al., 2009).

### Inter-Species QS Signals

#### A Widespread Communication Signal in Bacteria Kingdom: AI-2

Bacteria direct their behavior by sensing the environment. Although many of above AIs are highly specific being produced and recognized by a single species, new studies indicate that some molecules have the potential to enable inter-species communication (Bassler et al., 1997). QS-dependent genes of luminescence in *V. harveyi* stains can be activated by cell-free supernatant from cultures of several unrelated bacterial species (Bassler et al., 1997). The discovery of AI-2 was the first clear indication of inter-species signaling. The release of the activated methyl group from SAM to an acceptor molecule gives rise to S-adenosylhomocysteine (SAH), which is subsequently converted into S-ribosylhomocysterine (SRH) by the enzyme 5’-methylthioadenosine/S-adenosylhomocysteine nucleosidase (Pfs). SRH is cleaved by the enzyme LuxS to produce an unstable intermediate 4,5-dihydroxy-2,3-pentanedione (DPD), which then spontaneously cyclizes into the family of active AI-2 signaling molecules (Chen et al., 2002). Hydrophilic AI-2 is secreted by exporters, but the mechanism is unclear. Extracellular AI-2 can be recognized by three specific receptors (Pereira et al., 2013). LuxP is one of receptors, which is found in *Vibrionales.* It is a periplasmic binding protein and can interact with LuxQ, a membrane-spanning sensor protein, to form a two-component regulatory system, LuxPQ. Boron bound-AI-2 binds to LuxP, and induces LuxQ auto-phosphorylation as well as expression of QS-related genes (Miller et al., 2004). LsrB is the second receptor, a high-affinity substrate-binding periplasmic protein in *Salmonella typhimurium, Bacillus cereus*, and *Escherichia coli* (*E. coli*) (Pereira et al., 2009). Non-borated AI-2 is recognized by the LsrB receptor, and internalized by a transporter that composes of LsrC, LsrD, and the ATP-binding protein LsrA. After being phosphorylated by LsrK kinase, it becomes phosphor-AI-2 (P-AI-2). P-AI-2 binds to LsrR (a repressor of the lsr operon), promoting Lsr system expression (Pereira et al., 2009). Although the overall folding of LuxP and LsrB is similar, the sequence identity is only 11%. The third receptor is RbsB, proposed as AI-2 receptors in *A. actinomycetemcomitans* and *Haemophilus influenza* strain 86-028NP. These proteins have over 70% identity in homology with the periplasmic-binding component of the ribose ABC transporter in E. coli. However, due to the lack of crystal structures of RbsB-ligand complexes, the precise amino acid residues binding to AI-2 are unclear (Armbruster et al., 2011; Figure 1, inter-species QS signals part).

#### A Constantly Rediscovered Molecule: AI-3

Researchers have also purified a putative autoinducer signal, AI-3, from luxS/AI-2 bacterial enterohemorrhagic *E. coli* (Sperandio et al., 2003). Further studies have confirmed that AI-3 synthesis is independent of LuxS (AI-2 synthase) (Walters et al., 2006). A more recent study indicates that AI-3 are several products which belong to the pyrazinone family. These AI-3 molecules are formed via a series of reactions. Among those reactions, threonine dehydrogenase (Tdh) mediated AI-3 signal production and aminoacyl-tRNA synthetases-related spontaneous cyclization are two essential reactions (Kim et al., 2020). QseC receptor, a histidine kinase sensor found in *E. coli* and *V. cholerae*, senses AI-3 signals to regulate gene expression. It also senses the host hormones epinephrine (Epi)/norepinephrine (NE) (Moreira et al., 2010). However, AI-3 analogs have no effect on adrenergic signaling in human cells *in vitro* (Kim et al., 2020) (Figure 1, inter-species QS signals part).

#### An Important Metabolite Mediating Bacteria Communication: Indole

Over time, bacteria co-evolve with the host and thus play an important role in the host metabolic processes. Bacteria are indispensable for the metabolism of some nutrients in the host; especially amino acids and carbohydrates. In addition to the involvement of bacteria-derived metabolites in regulation of host physiology and the immune system, up-to-date evidence shows that they may work as a mode of communication that affects bacterial behavior in turn. Indole is a typical example of this model. It can impact activities of indole-producing and non-indole-producing bacteria in different ways (Lee and Lee, 2010). Indole is produced when tryptophan is degraded by tryptophanase (TnaA). However, environmental factors have a critical effect on this process. The tna operon expression is elevated at extracellular tryptophan-rich conditions. This operon transcribes TnaA and TnaB, which are the permeases responsible for environmental tryptophan uptake (Gong and Yanofsky, 2002; Figure 1, inter-species QS signals part). Indole production in cells reaches a maximum and is stably maintained in the stationary phase (Mueller et al., 2009). Carbon sources such as glucose, temperature, and pH also have direct effects on the concentration of extracellular indole (Lee and Lee, 2010). Without the requirement for specific receptor binding, indole molecules can activate succinate associated genes, or act on regulatory proteins with diverse biological functions such as spore or biofilm formation, drug resistance, virulence, and plasmid stability (Martino et al., 2003; Hirakawa et al., 2005). For instance, indole works on transcriptional regulator RamA to increase drug resistance in non-indole-producing *Salmonella enterica* and binds pyruvate kinase to induce spore formation in *Stigmatella aurantiaca*. However, the effect of indole on biofilm formation in *E. coli* strains is different among different studies (Martino et al., 2003; Lee et al., 2007; Zhang et al., 2007).

Some bacterial metabolites can be utilized by other bacteria or pathogens as a source of nutrients or an aid for colonization. These metabolites work as cues to affect gene expression that are responsible for virulence and invasion of pathogens. For example, short-chain fatty acids (SCFAs) promote the adhesion, flagellum growth, and virulence of specific bacteria via upregulation of the expression of related genes such as T3SS in *Salmonella typhimurium* invasion (Lawhon et al., 2002). Secondary bile acids (BAs) generated by *Clostridium scindens* are able to inhibit *C. difficile* colonization (Buffie et al., 2015). Collectively, there is no doubt that more QS signals will be found from metabolite molecules.

In gut, components of metabolites are much complex because host cells and bacteria involving. It is easy to confuse the QS signal and metabolites which both are productions of nutrients utilization and have some similar functions. Many QS signal syntheses depend on metabolites in the environment. Both AHLs and AI-2 are intermediates of SAM metabolism which is one part of methionine metabolism and methyl cycle. When cultured in an environment devoid of methionine, *E. coli* demonstrates a reduction in all components of the bacterial methyl cycle (Lin and Wang, 2017). The deficit of methyl cycle elements may further affect the production of QS signals. Carbon acyl chain is an integral part of AHLs structure with some acyl chains being derived from intermediates of fatty acid biosynthesis in the host (Papenfort and Bassler, 2016). Besides indole, a metabolite derived from tryptophan, signals in the AI-3 family mainly come from threonine dehydrogenized by threonine dehydrogenase. In general, recognizing metabolites-mediated QS signals is a process in which bacteria passively adapt the environment to survive. By contrast, the classical QS is much more like an evolutionary capability of bacteria to unite as a sociality.

## QS in the Gastrointestinal Tract

### QS May Contribute to Spatial Distribution of Intestinal Bacteria Under Physiological Condition

**FIGURE 2:**
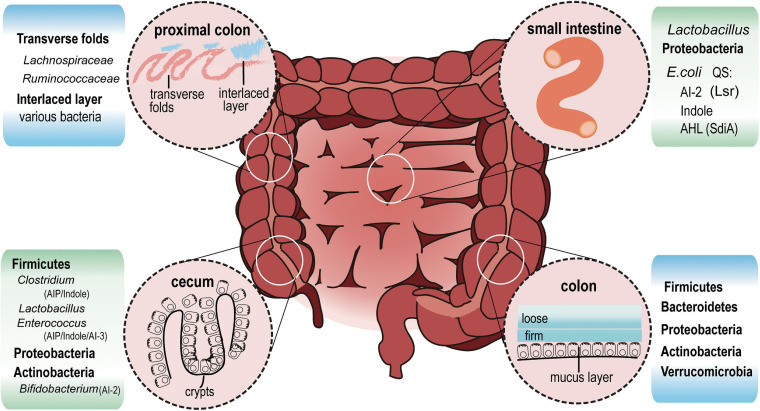
Intestinal bacterial distribution and QS signals. In the ileum (small intestine), *Lactobacillus* and Proteobacteria are the most abundant bacteria. *E. coli*, which belongs to Proteobacteria, communicates with other bacteria via AI-2, Indole and AHL signals. In cecum crypts, *Firmicutes* including *Clostridium*, *Lactobacillus*, *Enterococcus*, Proteobacteria, and Actinobacteria can be found. AIP, AHL, Indole, AI-2 and AI-3 may all exist in this region. At the distal end of the proximal colon, a thin but dense band called interlaced layer separates bacteria from epithelium. The interlaced layer of proximal colon is dominated by *Lachnospiraceae* and *Ruminococcaceae*. From transverse colon to rectum, mucus increases and divides into two layers. *Firmicutes*, *Bacteroidetes*, Actinobacteria, proteobacteria, and Verrucomicrobia live in the loose mucus layer.

Gut bacteria significantly affect the metabolic or immune status of the host (Hollister et al., 2014). In fact, the composition and relative abundance of gut bacteria is different along the length of gastrointestinal tract (Figure 2). QS is responsible for the production, extracellular accumulation, detection and response to AIs secreted by bacteria during a specific stage of growth (Winzer et al., 2002). Bacteria use QS signals as an active way to sense the environment and deliver information to its own community, as well as with other species. In the noisy environment of the gut, bacteria rely on QS to regulate survival and to compete with others for spatial dominance. However, the size of bacterial cell aggregates can influence QS signal response in turn. Smaller aggregates, but with more cells in each aggregate, showed higher bioluminescence intensity, secreted more AI-2, and had significantly higher QS-related gene expression, compared to the group with a larger aggregate but fewer cells in each aggregate (Gao et al., 2016). Another study combining micro-3D printing and scanning electrochemical microscopy revealed that QS-mediated communication can be observed in *Pseudomonas aeruginosa* within aggregates as small as 500 cells. However, at a defined distance of 8 μm, an aggregate containing more than 2,000 bacteria is required to stimulate QS in neighboring aggregates (Connell et al., 2014).

Based on these published studies, it can be deduced that the size of aggregates in crypts and transverse folds in the cecum and proximal colon is larger than that in the lumen where feces are constantly squeezed by intestinal motility. Species of *Firmicutes* living here may produce enough QS signals to influence its own community as well as neighbor species via AI-2 (Jakobsson et al., 2015). About 83.33% of *Firmicutes* contain LuxS protein orthologs, an essential synthase for AI-2 production, which is found in only 16.83% of *Bacteroidetes* (Thompson et al., 2015). This significant difference in AI-2 production capability might strengthen the competitive edge of *Firmicutes*, allowing them to dominate the cecum and proximal colon. Among *Bacteroidetes*, *B. thetaiotaomicron* acquire carbon sources either from dietary plant polysaccharides or mucus glycans in the absence of polysaccharides. They have high metabolic capacity and replicate themselves equally in mucus and lumen (Li et al., 2015). However, due to the lack of LuxS orthologs (KEGG base), they find it hard to compete with other species such as *Clostridia* and *Lactobacillus* etc., that take advantage of AI-2 for biofilm formation or self-growth (Lebeer et al., 2008).

Mucus-resident bacteria and luminal commensal bacteria are diverse, often being distributed in distinct niches where nutrients are utilized in different ways. *E. coli* residing in the outer mucus replicates quickly. In the intestinal lumen, *E. coli* remains in the stationary phase due to the limited glycoside hydrolase (Li et al., 2015). Mucus acts as a physical protective barrier to separate bacteria and the intestinal epithelium, and is constantly renewed. The inner mucus layer has fast turnover and ultimately gets converted into the loose outer mucus layer (Johansson, 2012). The outer layer is excreted with the colon contents by intestinal movement. The next round of food, accompanied by underlying mucus, allows bacteria to recolonize. AI-2 molecules regulate biofilm formation and allows adhesion and enrichment of some bacteria such as *Bifidobacterium* and *Lactobacillus* strains (Sun et al., 2014). Instead of recolonizing from luminal contents, *E. coli* cells living in mucus selectively proliferate during the mucus turnover period. In addition, AI-2 signal is implicated in iron metabolic regulation in bacteria including *Actinobacillus*, *Vibrio*, and *Bifidobacterium* (Fong et al., 2003; Kim and Shin, 2011; Li et al., 2011; Christiaen et al., 2014). Studies have reported that *E. coli* colonizing the mucus can utilize more iron than their lumen counterparts (Li et al., 2015), which might occur due to higher exposure to AI-2 signals in the mucus. QS signal may help commensal intestinal bacteria cooperate to strengthen the ability to resist colonization by invaders, and keep the dynamic balance by regulating relative abundance of certain species. This is certainly an area of research which requires further exploration.

### Communication via QS Between Intestinal Bacteria and Host Cells

Bacteria have co-evolved with their host for a long time. In humans, bacteria are crucial to maintain the epithelial barrier integrity and mucosal immune system via their components or bacteria-derived metabolites (Kayama et al., 2020). In addition to the classical way that microbiota influence host immune homeostasis by engaging pattern recognition receptors (PRR) at mucosal sites (Akira et al., 2006), pioneering research studies have revealed the possibility that intestinal bacteria interact with host cells by the QS system.

#### Bacteria-Derived QS Signaling Molecules Affect the Host Cells

**FIGURE 3:**
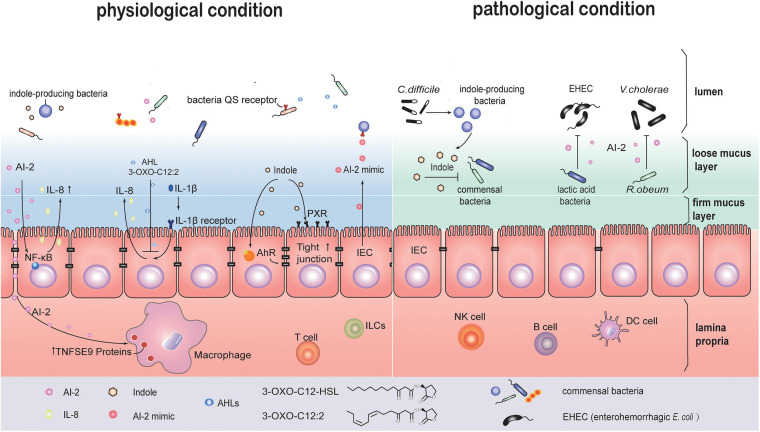
QS maybe involved in communication between intestinal bacteria and host cells under physiological or pathological conditions. In the physiological state, several QS signal molecules are implicated in the crosstalk between host cell and parasitic bacteria. AI-2 activates the NF-κB signaling pathway in IEC cells to upregulate the level of cytokine IL-8. AI-2 promotes TNFSF9 gene expression in macrophages. 3-oxo-C12:2 (AHLs) decreases IL-8 secretion in IL-1β-stimulated IECs. Indole enhances the epithelial barrier function via activating pregnane X receptor (PXR) and aryl hydrocarbon receptor (AHR). Meanwhile, IECs produce AI-2 mimic signals that can be recognized by AI-2 receptor, and regulate QS related genes expression in bacteria. Under pathological states, for example, *Clostridium difficile* infection, indole-producing bacteria sense QS signals and create a unique environment with high levels of indole, which inhibits common intestinal bacteria recovery and benefits *C. difficile* self-survival. “lactic acid bacteria” (LAB) such as *Bifidobacterium* can inhibit enterohemorrhagic *Escherichia coli* (EHEC) virulence by AI-2 signals. *Ruminococcus. obeum* restricts *Vibrio cholerae* colonization through AI-2 signals.

Early studies on bacterial infections reported that QS signals induce host cell apoptosis and can regulate secretion of immune mediators in independent ways. This suggests that QS signals may activate more than one signal transduction pathway in host cells (Shiner et al., 2006). In one study, crosstalk between AI-2 signaling and intestinal epithelial cells (IEC) was observed. Two strains of *E. coli*, BL21 and W3110, were co-cultured with the IEC line HCT-8 in a transwell system. These two strains are genetically similar, but the BL21 strain is capable of producing higher levels of extracellular AI-2 without expressing appropriate receptors to uptake AI-2 molecules. Transcription sequencing analysis of HCT-8 cells showed activation of NF-κB-mediated signaling pathways. The inflammatory cytokine, IL-8, was rapidly upregulated early, from 6 to 12 h, and then downregulated at 24 h (Zargar et al., 2015). AI-2, continuously secreted by different intestinal bacteria, especially those near the epithelia, can regulate negative-feedback of inflammatory response for immune tolerance. For example, TNFSF9 gene expression was significantly upregulated in AI-2 stimulated macrophages (Li et al., 2019). Indole can be recognized by aryl hydrocarbon receptor (AHR) and bind the pregnane X receptor (PXR) in IECs (Hubbard et al., 2015). This process may enhance epithelial barrier function by upregulating the expression of cell-junction-associated molecules, and also regulate epithelial cell differentiation from crypt stem cells (Shimada et al., 2013; Venkatesh et al., 2014; Figure 3).

#### Host Influence Bacteria Based on QS Signal Mimic

In addition to being regulated by bacterial QS molecules, host cells also respond to QS signaling by fighting back. When affected by bacteria-derived soluble molecules, IECs can secrete signal analogs that mimic AI-2, thus affecting gut bacteria. When LuxS mutant strains lacking AI-2 signal production were co-cultured with epithelial cells isolated from colon tissues, lsr gene transcription, usually induced by AI-2, was found to increase. This was attributed to an AI-2 mimic produced by IEC. Further, these signals were associated with epithelial tight-junction damages. The above findings suggest that the host-derived AI-2 mimic maybe associated with intestinal bacteria adhesion and epithelial barrier disruption (Ismail et al., 2016). In addition of the AI-2 mimic, bacteria can also make use of molecules secreted by the host as QS signals in the gut microenvironment. Host hormones such as catecholamines can promote bacterial growth. Epi/NE can be sensed by the QseC receptor of QS system (Clarke et al., 2006). More recent studies have shown other adrenergic receptors including QseE and CpxA that have significant differences in the phylogenetic profile with QseC also act as receptors (Reading et al., 2007; Karavolos et al., 2011; Karavolos et al., 2013). 1-octanoyl-rac-glycerol (OCL), a monoacylglycerol that is abundant in the mammalian gastrointestinal tract, forms triacylglycerols and works as a chemical chaperone placeholder to stabilize SdiA in *E. coli*, allowing its basal activity (Nguyen et al., 2015). Fatty acids (FAs), ubiquitously found in various organisms, have similar chemical structures with diffusible signal factors (DSF) family. DSFs are used by some Gram^–^ bacteria as QS signals for biofilm formation and virulence. FAs, as DSFs mimic, can inhibit the bacterial biofilm or other QS-dependent gene expression, and influence AHL and AI-2 signaling. The common pathogens inhabiting the human body, including *Burkholderia* spp., *P. aeruginosa*, *Vibro* spp., *Helicobacter pylori*, and *Salmonella*, utilize DSFs. Some of them specifically target the gastrointestinal system (Kumar et al., 2020). In the absence of physical barrier in small intestine, chemical barriers play a critical role in separating bacterial and epithelial cells in the small intestine and thus protecting the host against pathogen infection (Kayama et al., 2020). In addition to antimicrobial peptides and Reg3 family proteins (Kayama et al., 2020), FAs present in bile juice may also mimic QS signal to regulate bacterial biofilm formation (Kumar et al., 2020).

### Changes in Intestinal Bacterial Communication Under Pathological Conditions

*Pseudomonas aeruginosa* is a Gram^–^ opportunistic pathogen acting on human tissues. It acts though 3 main QS systems, including two AHL dependent LuxI/LuxR-type systems and a Pseudomonas quinolone signal (PQS) system. N-3-oxo-dodecanoyl-Lhomoserine lactone (3O-C12-HSL) and N-butyryl-L-homoserine lactone (C4-HSL) from the AHL signal family are used by *P. aeruginosa* to control more than 300 genes, many of which are involved in virulence regulation. The PQS system is associated with biofilm formation. When *P. aeruginosa* infects the human body, the above mentioned QS signals interact with human cells, leading to physiological and functional changes in immune cells including neutrophils, macrophages as well as epithelial cells (Holm and Vikström, 2014). Compared to a mutant *P. aeruginosa* strain lacking AHL production, the wild type strain containing 3O-C12-HSL and C4-HSL promoted macrophage phagocytosis. 3O-C12-HSL caused cell-volume increase which was associated with upregulation of Aquaporin 9 (AQP9), a chronic inflammation marker in inflammatory bowel disease (IBD) (Mesko et al., 2010; Holm and Vikström, 2014).

Subsequent research analyzed feces from healthy subjects and IBD patients with flares or under remission. AHLs were detected in these samples using liquid chromatography and mass spectrometry. Among AHLs, 3-oxo-C12:2 was significantly enriched in the healthy group and was higher in the IBD group under remission compared to IBD patients with flares. Unlike 3-oxo-C12 (3O-C12-HSL), 3-oxo-C12:2 could decrease IL-8 secretion in IL-1β-stimulated IECs, but had no effect on the epithelial paracellular permeability (Figure 2). Moreover, 3-oxo-C12:2 levels positively correlated with higher counts of *Faecalibacterium prausnitzii*, *Clostridium coccoides*, and *Clostridium Leptum* (Landman et al., 2018). These bacterial species are rare and belong to *Firmicutes*, especially Gram^–^ bacteria *F. prausnitzii*. In fact, an earlier study reported that oral administration of live *F. prausnitzii* or its supernatant could reduce the severity of 2,4,6-trinitrobenzenesulfonic acid (TNBS) colitis. The anti-inflammatory effects of *F. prausnitzii* are partly due to its secreted metabolites that block IL-8 production (Sokol et al., 2008).

There are high levels of indole and *Clostridium difficile* (*C. difficile*) toxin-induced QS signaling peptides in the feces from *C. difficile* infection (CDI) patients compared with CDI-negative diarrhea patients. It suggests that *C. difficile* uses QS signals to regulate its infection in the gastrointestinal tract, and this QS signal is related with indole production. However, *C. difficile* lacks the tryptophanase gene, which aids in indole production. Subsequent findings indicate that *C. difficile* may use toxin-induced QS signals to regulate the relative abundance of indole-producing bacteria and create a conducive environment for their survival (Figure 2). Meanwhile, commensal gut bacteria with a lower indole tolerance than *C. difficile* would be inhibited from recovering, resulting in a more beneficial environment for the colonization and expansion of *C. difficile* (Darkoh et al., 2019).

The production of AI-2 has a broad impact on the relative abundance of two dominant phyla of bacteria in the gut. Researchers found the abundance of *Firmicutes* phyla in mice can be significantly reduced by streptomycin treatment for 28 days. Consequently, *Bacteroidetes* become dominant and its relative abundance reaches approximately 90%. Additionally, three types of mutant *E. coli* are mono-colonized in those antibiotic-treated mice. One mutant strain lacks the AI-2 kinase gene lsrK, which allows extracellular AI-2 accumulation. Depletion of lsrR, a repressor of lsrK transcription, promotes another strain to deplete environmental AI-2 by internalizing them. The third mutant strain has no effect on AI-2 production and accumulation due to the removal of signal synthase LuxS. The three mutant strains could be assembled into three groups, namely ΔlsrK AI-2 secreting-, ΔlsrRΔLuxS AI-2 absorbing- and ΔlsrKΔLuxS neutralizing- group. The ΔlsrK AI-2 secreting-group showed an increasing ratio of *Firmicutes* to *Bacteroidetes* (Thompson et al., 2015).

### The Role of QS System in the Gut Microbiome Community Regulated by Probiotics

Probiotics, made up of “good bacteria,” help reverse gut dysbiosis. The QS system in the gut microbiome can be regulated by probiotics. In rodent models, *E. coli* competes with *Salmonella typhimurium* (*S. typhimurium*) for nutrient iron, resulting in reduction of *S. typhimurium* colonization (Deriu et al., 2013). LuxS gene plays a role in motility and virulence of *S. typhimurium* (Choi et al., 2007). However, AI-2 signals secreted by *S. typhimurium* for invasion are inhibited by *E. coli*. *Bifidobacterium*. belonging to “lactic acid bacteria” (LAB), contains the luxS gene and can therefore produce AI-2 molecules to regulate biofilm formation (Sun et al., 2014). Similarly, in weaning swine, the virulence of enterohemorrhagic *E. coli* (EHEC) is inhibited by the presence of AI-2 signals produced by LAB (Kim et al., 2018; Figure 3).

In a study about *V. cholerae* infection in Bangladesh, researchers collected feces from three cohorts including cholera patients, healthy adults and healthy children. The time-series metagenomic analysis suggested that accumulation of certain bacterial taxa is associated with recovery from a *V. cholerae* infection. In gnotobiotic mice, bacterial community transplantation induces increase in *Ruminococcus obeum* relative abundance, however *V. cholerae* colonization is restricted. LuxS gene expression and AI-2 production in *R. obeum* increase with *V. cholerae* invasion. These molecules target other pathways to inhibit *Vibrio* pathogenicity, irrespective of whether a LuxP sensor exists or not (Hsiao et al., 2014). This study indicates an unknown, novel function of the QS system that is different from the canonical pathways discussed previously (Figure 3).

## QS as a Prospective Therapeutic Target for Microbial Infections

Novel therapeutic strategies can be exploited by targeting the bacterial QS system. Higher AI-2 levels were observed in colon mucosa and stools from colorectal adenoma and colorectal cancer (CRC) patients compared to healthy individuals. Importantly, AI-2 concentration was found to increase with CRC progression, suggesting its potential as a novel marker for CRC clinic screening. Another recent research on burn-site infections focused on the *P. aeruginosa* QS system. Burn injury is often accompanied with gut microbiome dysbiosis, impaired intestinal integrity, immune dysregulation and bacterial extra-intestinal translocation. *P. aeruginosa* is the main pathogen in post-burn infection. Its toxic products can prolong intestinal dysfunction and aggravate systemic infection. Researchers observed an improvement in intestinal dysfunction and a decrease in *P. aeruginosa* dissemination after inhibiting MvfR transcriptional factor, an important QS-associated transcriptional regulator controlling the virulence of this strains, using a MvfR antagonist (Adiliaghdam et al., 2019). In fact, antibacterial therapeutic strategies have started to expand from antibiotics usage to developing inhibitors based on QS systems such as anti-virulence or anti-biofilms. These inhibitors or analogs exist endogenously either in bacterial or eukaryotic cells. An example is Carnosic acid, a specific inhibitor of the QS system in *S. aureus*. It is found in rosemary leaves and can inhibit Agr expression and *S. aureus virulence* at a low concentration (Nakagawa et al., 2020). Taken together, these thrilling studies point to a new potential of drug development for refractory infectious diseases.

## Conclusion and Further Perspectives

Researches in the field of bacterial QS have expanded rapidly. QS has a critical role in bacterial behavior during adaption and development in diverse environments like the human gut. It has the potential to affect infection status and disease development though bacterial-host crosstalk. Although the fundamental biology of bacterial sociality is complex, we have come a long way in our attempt to elucidate the roles and functions of QS in microbial communities. In addition, we have also realized the significance of understanding the interactions between microbes and their host.

Many critical questions still exist. In gut, symbiotic bacteria occupy in different areas along the gastrointestinal (GI) tract of host. Besides the influence of GI microenvironment on symbiotic bacteria occupation, it is still lack of knowledge about how QS signal systems participate in their occupation and interact with host cells. Addressing this point would be useful in exploring new methods to strengthen resistibility of commensal intestinal bacteria to colonization by invaders and maintain mucous stability in host. In addition, despite the discovered important roles of QS signals in bacterial communication, it will probably take more years to address the related molecules mechanisms in details. In the respective of problematic antibiotic resistance, the development of alternative therapies for infectious diseases is one of the major societal challenges at this moment. Probiotics has been recognized as a method of treatment. Fecal transplantation (FMT) recently drives more attention for many diseases, particularly in gastrointestinal disorders and metabolic diseases (He et al., 2017; Ding et al., 2019; Antushevich, 2020). Anti-virulence therapy by using various quorum-sensing inhibitors or agents that interfere with QS in bacterial pathogens currently is also a reasonable and promising strategy. Although we have obtained some positive results regarding to QS inhibitors in previous studies, it is still a long way to go in identifying the molecular targets of these potential agents and speeding their application in the clinic. Exploring new applications of available drugs used in the clinic for other diseases probably will be an approach to save time and money.

With the development of high-throughput-omics techniques, scientific cognition of QS signals and their regulating pathways can be clarified. Sorting out fundamental differences between diverse QS systems will give us an opportunity to understand the biology of bacterial sociality better. Moreover, it will give us the confidence to translate QS studies for potential therapeutic development for devastating infectious diseases.

## Author Contributions

LW wrote the article. YL revised the manuscript. Both authors researched data for the article, made substantial contributions to the content, and edited the manuscript.

## Conflict of Interest

The authors declare that the research was conducted in the absence of any commercial or financial relationships that could be construed as a potential conflict of interest.
